# Association between problematic TikTok use and mental health: A systematic review and meta-analysis

**DOI:** 10.3934/publichealth.2025027

**Published:** 2025-04-16

**Authors:** Petros Galanis, Aglaia Katsiroumpa, Zoe Katsiroumpa, Polyxeni Mangoulia, Parisis Gallos, Ioannis Moisoglou, Evmorfia Koukia

**Affiliations:** 1 Clinical Epidemiology Laboratory, Faculty of Nursing, National and Kapodistrian University of Athens, Athens, Greece; 2 Faculty of Nursing, National and Kapodistrian University of Athens, Athens, Greece; 3 Faculty of Nursing, University of West Attica, Athens, Greece; 4 Faculty of Nursing, University of Thessaly, Larissa, Greece; 5 Laboratory Nursing Counselling, Faculty of Nursing, National and Kapodistrian University of Athens, Athens, Greece

**Keywords:** TikTok, mental health, problematic use, addiction, anxiety, depression, systematic review, meta-analysis

## Abstract

**Background:**

TikTok is a significant part of social media usage, since 25.6% of the total global population has a TikTok account, and, thus, scholars should pay attention to its association with users' mental health.

**Objective:**

To synthesize and evaluate the association between problematic TikTok use and mental health.

**Methods:**

We applied the Preferred Reporting Items for Systematic Reviews and Meta-Analysis guidelines in our review. The review protocol was registered with PROSPERO (CRD42024582054). We searched PubMed, Scopus, Web of Science, PsycINFO, ProQuest, and CINAHL until September 02, 2024.

**Results:**

We identified 16 studies with 15,821 individuals. All studies were cross-sectional and were conducted after 2019. Quality was moderate in 10 studies, good in three studies, and poor in three studies. Our random effects models showed a positive association between TikTok use and depression (*β* = 0.321, 95% confidence interval: 0.261 to 0.381, *p* < 0.001, *I^2^* = 78.0%, *n* = 6 studies), and anxiety (*β* = 0.406, 95% confidence interval: 0.279 to 0.533, *p* < 0.001, *I^2^* = 94.8%, *n* = 4 studies). Data to perform meta-analysis with the other mental health variables were limited. However, our descriptive data showed a positive association between TikTok use and body image issues, poor sleep, anger, distress intolerance, narcissism, and stress.

**Conclusion:**

Our findings suggest that problematic TikTok use has a negative association with several mental health issues. Given the high levels of TikTok use, especially among young adults, our findings are essential to further enhance our understanding of the association between TikTok use and mental health. Finally, there is a need for further studies of better quality to assess the association between problematic TikTok use and mental health in a more valid way.

## 1. Introduction

In 2024, approximately 64% of the total global population was a social media user, while this percentage is predicted to approach 74.4% by 2028 [1]. TikTok is a significant part of social media usage, since 25.6% of the total global population has a TikTok account. At least 26.3% of the 4 billion people who use social media every month use TikTok once a month. In fact, TikTok was downloaded more than 730 million times in 2023 alone, more than any other social media platform. Globally, the average time spent on TikTok was 95 minutes per day, more than any other social media (Instagram: 62 minutes, X [Twitter]: 30 minutes, Snapchat: 19 minutes) [2]. TikTok is one of the fastest growing social media platforms in the world, having grown from 347 million users in 2018 to 1.4 billion users in 2021 and 2.1 billion users in 2024. Moreover, the number of TikTok users is expected to reach 2.4 billion by the end of 2029. For instance, TikTok had around 170 million users in the USA in 2024, representing 49.1% of the total population size [1]. TikTok is particularly popular among adolescents and young adults, since about 64% of TikTok users are between 16 and 29 years old. For instance, 33% of the USA users are younger than 19 years old. There is a slight predominance of the male gender among TikTok users, as the percentage of men is 54.8% and women is 45.2% [1],[2].

The COVID-19 pandemic stands out as the first global health crisis to unfold in an era characterized by social media and unparalleled connectivity. Throughout this period, social media usage surged worldwide. During the COVID-19 pandemic, social media platforms played a crucial role in shaping global responses and adaptations [3]. This importance was underscored by a notable surge in social media activity, with Facebook experiencing nearly a 30% increase in traffic in early 2020 following the WHO declaration of the pandemic [4],[5]. More broadly, there was a 9.9% rise in the global number of social media users [6], indicating a growing reliance on social media for connectivity during this health crisis [7]. Social media not only allowed individuals in isolation to stay in touch with friends and family but also facilitated communication with healthcare professionals [8]. The abrupt transition to a digital environment and increased screen dependency had a profound impact on people during the pandemic. Lockdowns and social isolation resulted in a significant rise in daily screen time [9],[10]. Furthermore, social media played a crucial role in maintaining a steady flow of information, thereby enhancing and disseminating evidence by linking individuals and communities. For example, analyses of Twitter activity in Italy during the pandemic revealed both positive and negative outbreaks from tweets [11]. Additionally, Twitter data provided early-warning signs of COVID-19 outbreaks in Europe during the winter of 2019–2020, even before local infection sources were publicly announced [12]. As the pandemic unfolded, social media quickly became an essential tool for generating, sharing, and consuming information. In summary, a scoping review pinpointed five significant public health themes concerning the function of online social media platforms during the COVID-19 pandemic. These themes encompassed tracking public opinions, identifying infodemics, assessing mental health, detecting or forecasting COVID-19 cases, evaluating governmental responses to the pandemic, and examining the quality of health information in educational prevention videos [13].

TikTok is a social media application that enables users to edit and post short videos using tools provided by the platform including music, filters, and special effects [14]. Also, TikTok users may interact with original videos. TikTok users may have access to a section with a feed of videos posted by followers, and a section with a feed of videos posted by strangers which are selected by TikTok's artificial intelligence machine according to the user's activity [15]. Since the TikTok artificial intelligence machine is extremely sophisticated, users are exposed to a large amount of video content that is presumably personally relevant and highly engaging [16],[17]. In this context, TikTok users are usually exposed to videos that are posted by users one does not know personally [18]. Thus, TikTok is significantly different from other social media applications, and scholars should focus on the impact of this highly popular social media application on users' health and well-being. To maintain visibility on TikTok, content creators must adhere to the platform's community guidelines to prevent their content from being flagged and removed. For example, TikTok explicitly prohibits the display, promotion, or sharing of content related to suicide, self-harm, disordered eating, and dangerous weight loss practices [19]. However, creators can outsmart the platform's algorithm by modifying words and phrases in their text descriptions. Common tactics include using abbreviations, replacing letters with symbols (e.g., “self-h@rming” instead of “self-harming”), and substituting entire words or phrases (e.g., “unaliving” in place of “suicide”). One common example is creators referring to their non-suicidal self-injury scars or wounds as “barcodes” instead of explicitly mentioning scars [20]. TikTok users frequently view videos from unknown content creators, increasing their likelihood of encountering harmful content due to a lack of knowledge about these creators' backgrounds and intentions. Specifically, TikTok may expose users to potentially damaging content related to self-harm, eating disorders, sexual assault, fat-shaming, and self-harm. Consequently, users' feeds are flooded with distressing and harmful content, which can have a considerable cumulative effect on their mental well-being [21],[22]. The association between problematic social media use and mental health is well established since several systematic reviews and meta-analyses have focused on this issue [23]–[30]. However, no systematic review up to now has investigated the association between problematic TikTok use and mental health. Systematic reviews have focused up to now on the association between Facebook, Instagram, Twitter, MySpace, Internet, social media in total, and mental health variables. Briefly, the mental health variables that have been investigated in systematic reviews included anxiety, depression, psychological distress, well-being, life satisfaction, narcissism, eating disorders, and sleep disturbances. The vast majority of these reviews focused on the association between problematic social media use and depression and anxiety. More specifically, five systematic reviews identified a positive association between problematic social media use and depression [23]–[26],[29], while five reviews identified a positive association between problematic social media use and anxiety [23]–[25],[29],[30]. Additionally, literature showed that problematic social media use is associated with increased sleep disturbances [24],[25], increased psychological distress [25],[29], and reduced well-being [24]. Among social media platforms, only Facebook has been studied through systematic reviews and meta-analyses. Research suggests the negative association between problematic Facebook use and well-being [28], and the positive association between problematic Facebook use and sleep disturbances [28], as well as narcissism [27].

Although several studies [31]–[34] have examined the association between problematic TikTok use and mental health there is no systematic review to summarize the evidence on this field. Recently, a systematic review examined the impact of TikTok on adolescents' mental health [35]. This review underscored four main themes related to TikTok usage: its overall influence on adolescents' mental health, the risk of problematic use and behavioral addiction, impacts on body image and self-esteem, and the potential for promoting behaviors linked to mental illness. The authors voiced concerns about TikTok's possible negative effects on teenagers, such as decreased life satisfaction, an increased risk of “contagion” of certain psychiatric symptoms, and patterns of problematic use. However, this review assessed only studies including adolescents. Moreover, authors did not perform a meta-analysis to summarize their findings.

By aggregating primary studies, meta-analysis enhances the sample size and consequently boosts the ability to examine the effects of interest, offering a precise estimation of these effects [36]. The data derived from meta-analyses are more advantageous than those from conventional systematic reviews. In meta-analysis, decisions are made transparently, and statistical analysis provides an objective assessment of the combined quantitative evidence [37]. This systematic and transparent approach in meta-analysis aids in resolving discrepancies and uncertainties among studies, leading to significant conclusions. In brief, meta-analysis increases statistical power through increased sample size, allows for an objective appraisal of evidence, identifies sources of heterogeneity between studies, resolves uncertainty when studies disagree, and establishes questions for future studies [38],[39].

TikTok seems to cause higher levels of addiction than other social media, and, thus, special attention should be given on this app. In particular, the endless scrolling feature and the unpredictable rewards on TikTok likely enhance the app's addictive nature by potentially creating a flow-like experience for users, marked by intense concentration and efficiency in their activity. When users enter this flow-like state, they may lose track of time, unaware of how long they have been engaged [40]. Additionally, the TikTok interface is simple and intuitive, with only a few buttons and sections to navigate, which further facilitates entering this “flow” [41]. The artificial intelligence-generated personalized “For You” feed is also a significant factor in TikTok addictive appeal. Unlike other social media platforms, the TikTok feed is not curated based on users' explicit content preferences. Instead, artificial intelligence suggests content and evaluates users' interactions, such as likes, comments, and shares, to refine future content suggestions, creating a self-reinforcing cycle that becomes more precise with continued use. These in-app features extend the duration users spend on TikTok, thereby increasing the platform's addictive potential. To enhance this effect, developers frequently update TikTok's layout and introduce new features, encouraging users to spend more time exploring and adapting to the changes [17].

Thus, our aim was to perform a systematic review and meta-analysis to examine the association between problematic TikTok use and mental health. To the best of our knowledge, this is the first systematic review and meta-analysis that examined the association between problematic TikTok use and mental health. Considering the advantages of meta-analysis, our work would offer scholars a more precise estimation of the association between problematic TikTok use and mental health variables. Additionally, our meta-analysis may identify possible sources of heterogeneity between studies, and may offer new ideas for future studies on this field.

We should notice that in our study, the definition of problematic TikTok use was based on its measurement with valid tools such as the “Bergen Social Media Addiction Scale” (BSMAS) [42],[43], which measures the level of TikTok addiction among users, and, thus, problematic TikTok use. Developers of the BSMAS identify six components of addiction such as salience, tolerance, mood modification, relapse, withdrawal, and conflict [44]–[46]. In brief, “salience” refers to excessive preoccupation with TikTok, “tolerance” refers to users' need to use social media more and more to be satisfied, “mood modification” refers to the fact that social media may improve users' mood, “relapse” refers to users' failure to cut down the social media use, “withdrawal” refers to the existence of negative emotions when social media usage is stopped, and “conflict” refers to the fact that social media usage may have a negative impact in everyday activities. Furthermore, considering that increased time spent on TikTok could be indicative of problematic TikTok use, we included studies that measured the time spent on TikTok.

Moreover, regarding mental health, we considered the following variables in our systematic review: anxiety, anxiety disorders, depression, distress, eating disorders, emotional disorders, sleep disorders, loneliness, self-esteem, life satisfaction, and well-being. In particular, “anxiety” refers to anticipation of a future concern and is more associated with muscle tension and avoidance behavior. Individuals that experience intense and excessive fear and worry may suffer from anxiety disorders. There are several types of anxiety disorders including generalized anxiety disorder, social anxiety disorder, and panic disorder [47]. Individuals with depression feel extreme sadness for excessive time. Moreover, depressed people loss their interest for long period of time [48]. “Distress” is defined as an unpleasant, negative state in which coping and adaptive mechanisms are unable to recover a person to physiological and/or psychological balance [49]. Behavioral problems known as eating disorders are defined by significant and ongoing disruptions in eating patterns, together with the accompanying distressing thoughts and feelings [50]. The term “emotional disorders” refers to a group of long-term, frequently repeated psychological disorders that are linked to serious impairments in productivity, interpersonal functioning, and quality of life [51]. Problems with the quantity, timing, and quality of sleep are known as sleep disorders. Sleep disorders cause distress during the day and functional impairment [52]. Loneliness is an unpleasant emotional response to perceived isolation [53]. Self-esteem is the belief in one's own value, skills, or abilities [54]. Life satisfaction is a measure of an individual's quality of life and can be evaluated in terms of self-concepts, relationship satisfaction, mood, and goals attained [55]. The combination of feeling and performing well is known as well-being [56].

## 2. Materials and methods

### 2.1. Data sources and strategy

We applied the Preferred Reporting Items for Systematic Reviews and Meta-Analysis (PRISMA) guidelines [57] to perform our systematic review and meta-analysis. The review protocol was registered with PROSPERO (CRD42024582054).

We searched PubMed, Scopus, Web of Science, PsycINFO, ProQuest, and CINAHL from inception to September 02, 2024. We used the following strategy in all fields: [(“problematic use” OR addiction* OR disorder*) AND (TikTok OR “short-form video*” OR “short video*” OR reel*)]AND (anxiety OR depression OR distress OR stress OR loneliness OR self-esteem OR “life satisfaction” OR well-being OR sleep OR “mental health” OR “mental issue*” OR “eating disorder*” OR “emotional disorder*” OR “anxiety disorder*”).

### 2.2. Selection and eligibility criteria

First, we removed duplicates and then we screened titles and abstracts. Finally, we screened full texts. Also, we examined the references of all articles included in our review. In particular, two independent authors performed study selection, and a third senior author resolved the discrepancies. We assessed inter-rater reliability between two authors by using Cohen's kappa [58]. Agreement was very good since Cohen's kappa was 0.82 [59].

We applied the following inclusion criteria: (a) studies that investigated the association between TikTok use and mental health issues, (b) studies that measured levels of problematic TikTok use with valid tools, such as the BSMAS that measures levels of TikTok addiction, (c) studies that measured time spent on TikTok since we considered that increased time spent could be indicative of problematic TikTok use, (d) quantitative studies, (e) studies that included individuals without mental disorders, (f) articles published in English, and (g) articles published in journals with a peer review system. We did not apply criteria regarding study population, e.g., gender, age, race, and ethnicity. We excluded studies that investigated any short-form video applications and not specifically TikTok. Also, we excluded studies that specifically sampled participants from a clinical sample, such as patients with depression and eating disorders. Moreover, we excluded meeting or conference abstracts, case reports, qualitative studies, reviews, meta-analyses, protocols, editorials, and letters to the Editor.

### 2.3. Data extraction

Two scholars independently extracted the following data from each study: first author, year of publication, location, data collection time, sample size, age, percentage of females, study population (adults, university students, or school students), study design, sampling method, response rate, scales for the assessment of TikTok use, mental health variables, scales for the assessment of mental health variables, unstandardized regression coefficients (betas) from linear regression models with mental health variables as the dependent variables, correlation coefficients between TikTok use and mental health variables, other measures of effect (e.g., odds ratio, mean score difference), 95% confidence intervals (*CI*s) for the measures of effects, and *p*-values.

### 2.4. Quality appraisal

We applied the Joanna Briggs Institute (JBI) critical appraisal tools [60] to assess the quality of the studies. Since all studies in our systematic review were cross-sectional, we applied the tool for this type of study. The JBI tool examines eight dimensions of potential bias. In brief, the tool assesses the description of inclusion criteria, description of study settings, measurement of exposure and outcome, identification and elimination of confounders, and methods of statistical analysis. In cross-sectional studies, a score of 7–8 points indicates good quality, a score of 4–6 points indicates moderate quality, and a score ≤3 indicates poor quality. Two independent reviewers applied the JBI tool in our review, and a third senior author resolved the differences.

### 2.5. Statistical analysis

For each study, we extracted measures of effect (e.g., unstandardized regression coefficients, correlation coefficients, and odds ratios), standard errors for measures of effect, 95% *CI*s for measures of effect, and *p*-values. To examine the association between TikTok use and mental health, we transformed unstandardized regression coefficients and other measures of effect into standardized regression coefficients (*β*) [61]. Unstandardized regression coefficients may be in different measurement units or scales, and, thus, direct comparison may be meaningless. Also, comparison between different measures of effect is impossible in meta-analysis. Moreover, standardized regression coefficients allow comparability since we can compare the impact of different predictor variables on the outcome variable, regardless of their original units. In this way, standardized regression coefficients allow us to identify which predictor variable has the strongest or weakest influence. The direct pooling of unstandardized regression coefficients is not meaningful across studies. We must express explanatory variables similarly in order to pool their effects when measured using different scales. The standardized regression coefficient might provide a way to summarize the results in this situation. The estimate obtained from an analysis of variables that have been normalized so that their variances and standard deviations equal one is known as the standardized regression coefficient. Thus, the number of standard deviations that the outcome variable will vary for every standard deviation that the explanatory variable increases is known as the standardized regression coefficient. Thus, we used standardized regression coefficients to eliminate these problems and combine studies with different measurement units or scales in a more meaningful and straightforward way. Higher absolute values of standardized regression coefficients indicate higher association between the independent variable and the outcome variable [61],[62].

We calculated the pooled standardized regression coefficient and 95% *CI*. We measured heterogeneity between studies with the *I^2^* statistic and the *p*-value for the Hedges *Q* statistic. *I^2^* values higher than 75% indicate high heterogeneity. Also, a *p*-value < 0.1 for the Hedges *Q* statistic indicate statistically significant heterogeneity [63]. The heterogeneity between results was very high, and, thus, we applied the random effects model to calculate the pooled standardized regression coefficient. Among mental health variables sufficient data to perform meta-analysis were available only for depression (six studies) and anxiety (four studies). For all other mental health variables data were available for ≤2 studies; distress (*n* = 2), loneliness (*n* = 2), narcissism (*n* = 1), body image issues (*n* = 1), poor sleep (*n* = 1), life satisfaction (*n* = 1), disordered eating behavior (*n* = 1), anger (*n* = 1), and self-esteem (*n* = 1). We conducted a leave-one-out sensitivity analysis to examine the influence of each study on the pooled standardized regression coefficient. A priori, we considered location, data collection time, sample size, age, percentage of females, population, study design, sampling method, response rate, and quality of studies as possible sources of heterogeneity. Due to the limited variability of these variables and the limited number of studies, we performed meta-regression analysis for data collection time, sample size, age, and percentage of females. Also, we performed subgroup analysis for the quality of the studies. We used the funnel plot and the Egger's test to assess the publication bias. A *p*-value < 0.05 for Egger's test and the asymmetry of the funnel plot indicate the presence of publication bias. We used OpenMeta [Analyst] to perform our meta-analysis [64].

## 3. Results

### 3.1. Identification and selection of studies

**Figure 1.**
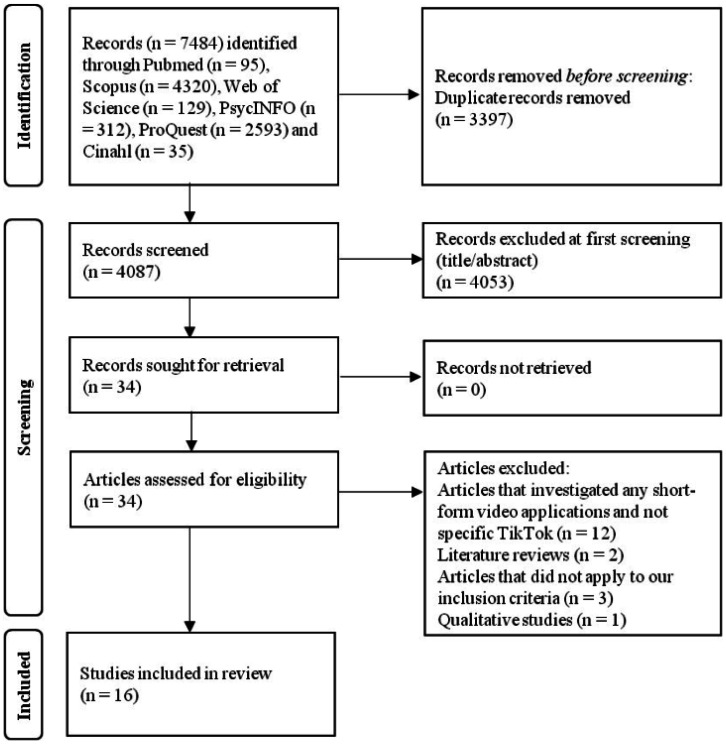
Flowchart of our literature search.

After an initial search, we found a total of 7484 records. After removal of duplicates, 4087 records were left. Then, we excluded 4053 records through title/abstract screening. We reviewed 34 articles. Applying our inclusion/exclusion criteria, we included 16 articles in our review (Figure 1).

### 3.2. Characteristics of the studies

Table 1 presents the main characteristics of the 16 studies included in our systematic review. A total of 15,821 individuals were included in our review. Seven studies were conducted in Asia [China (*n* = 3), Pakistan (*n* = 2), Saudi Arabia (*n* = 1), and Turkey (*n* = 1)] [31],[65]–[70], four studies in Europe [United Kingdom (*n* = 1), France (*n* = 1), Poland (*n* = 1), and Spain (*n* = 1)] [32],[33],[71],[72], three studies in America [USA (*n* = 2) and Honduras (*n* = 1)] [34],[73],[74], one study in Oceania [Australia] [75], and one study in Africa [Nigeria] [76].

One study collected their data in 2019 [74], two studies in 2020 [68],[72], one study in 2021 [71], six studies in 2022 [31],[33],[67],[69],[70],[73], five studies in 2023 [32],[34],[65],[75],[76], and one study [66] did not report the data collection time.

Eight studies included university students [31],[32],[34],[66],[69],[73],[75],[76], three studies included adults [33],[70],[72], and four studies included school students [65],[67],[68],[74]. Mean age ranged from 14.0 years to 33.8 years. Percentage of females ranged from 42.0% to 100.0%. There was a higher percentage of female participants in 11 out of the 12 studies. All studies were cross-sectional and used a convenience sampling method.

### 3.3. Measurement scales for TikTok use and mental health variables

Table 2 shows the assessment of TikTok use and mental health variables in the studies that were included in our review. Seven studies used a valid scale to measure TikTok use [31]–[34],[68]–[70]. In particular, five studies [31]–[34],[69] used an adapted version of the Bergen Social Media Addiction Scale by replacing “social media” with “TikTok” throughout the scale. Also, two studies [68],[70] used an adapted version of the Smartphone Addiction Scale-Short Version (SAS-SV) by replacing “smartphone” with “TikTok” throughout the scale. Nine studies [65]–[67],[71]–[76] did not use a valid tool to measure TikTok use but they used simple items (e.g., How often do you use TikTok?, Do you use TikTok?).

Studies assessed 13 mental health variables: depression, anxiety, stress, distress intolerance, loneliness, anger, narcissism, self-esteem, body image issues, poor sleep, life satisfaction, disordered eating behavior, and disordered eating. Two studies [74],[76] did not use valid scales to measure mental health variables. Depression was assessed with the Patient Health Questionnaire-9 (PHQ-9), the Center for Epidemiologic Studies Depression Scale (CES-D scale), and the Depression Anxiety Stress Scales-21 (DASS-21). Anxiety was assessed with the Social Interaction Anxiety Scale (SIAS), the Generalized Anxiety Disorder-7 (GAD-7), the State-Trait Anxiety Inventory (STAI), and the DASS-21. Stress, distress intolerance, loneliness, anger, narcissism, poor sleep, life satisfaction, disordered eating behavior, and disordered eating were assessed with the DASS-21, Distress Intolerance Scale (DIS), UCLA Loneliness Scale, Adolescent Anger Rating Scale (AARS), Narcissistic Personality Inventory-16 (NPI-16), Pittsburgh Sleep Quality Index (PSQI), Satisfaction With Life Scale (SWLS), Eating Attitudes Test-26 (EAT-26), and Sick, Control, One, Fat, Food (SCOFF), respectively.

### 3.4. Quality assessment

Quality was moderate in 10 studies [31],[65],[67],[69]–[75], good in three studies [32]–[34], and poor in three studies [66],[68],[76] (Supplementary Table S1). The main threats to study quality were the non-valid assessment of TikTok use, and the failure to identify and eliminate confounding factors.

### 3.5. Meta-analysis

#### 3.5.1. Depression

**Figure 2.**
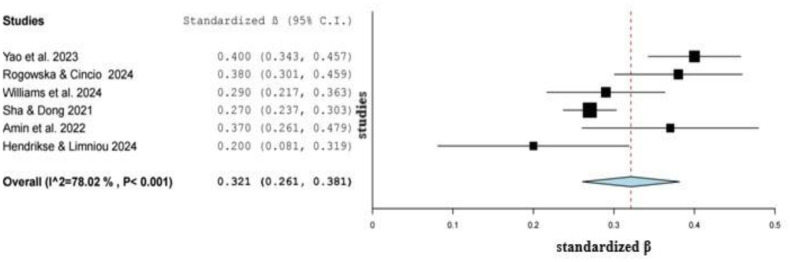
Forest plot of the association between TikTok use and depression.

We calculated the standardized regression coefficients from unstandardized regression coefficients and other measures of effect referring to the relationship between TikTok use and depression. In particular, we calculated standardized regression coefficients from data in six studies [31]–[34],[68],[70]. We found a statistically significant positive association between TikTok use and depression since the pooled standardized regression coefficient was 0.321 (95% *CI*: 0.261 to 0.381, *p* < 0.001) (Figure 2). Standardized regression coefficients between studies ranged from 0.200 (95% *CI*: 0.081 to 0.319) to 0.400 (95% *CI*: 0.343 to 0.457). All studies found a statistically significant positive association between TikTok use and depression. Heterogeneity between results was high (*I^2^* = 78.0%, *p*-value for the Hedges *Q* statistic < 0.001). A leave-one-out sensitivity analysis showed that no single study had a disproportional effect on the pooled standardized regression coefficient, which varied between 0.302 (95% *CI*: 0.247 to 0.356, *p* < 0.001), with Yao et al. (2023) [70] excluded, and 0.338 (95% *CI*: 0.275 to 0.401, *p* < 0.001), with Hendrikse and Limniou (2024) [32] excluded (Supplementary Figure S1). The *p*-value for Egger's test (0.97) and symmetry of the funnel plot (Supplementary Figure S2) indicated an absence of publication bias.

Meta-regression analysis showed that increased age was associated with an increased pooled standardized regression coefficient (*β* = 0.012, *p* < 0.001). Moreover, the pooled standardized regression coefficient was independent of the data collection time (*β* = 0.006, *p* = 0.76), sample size (*β* = −0.001, *p* = 0.25), and percentage of females (*β* = −0.001, *p* = 0.77).

Subgroup analysis showed that the positive association between TikTok use and depression was stronger among studies with a moderate and high risk of bias (pooled *β* = 0.34, 95% *CI*: 0.24 to 0.44, *I^2^* = 87.6%) than studies with a low risk of bias (pooled *β* = 0.29, 95% *CI*: 0.21 to 0.39, *I^2^* = 69.6%).

**Table 1.** Main characteristics of the studies included in this systematic review.

**Reference**	**Location**	**Data collection time**	**Sample size (*n*)**	**Age, mean (*SD*)**	**Females (%)**	**Population**	**Study design**	**Sampling method**	**Response rate (%)**
[70]	China	2022	822	27.5 (5.9)	65.3	Adults	Cross-sectional	Convenience sampling	58.1
[31]	Pakistan	2022	240	18–25 years; 87%, 26–32; 13%	42.0	University students	Cross-sectional	Convenience sampling	NR
[66]	Pakistan	NR	350	NR	NR	University students	Cross-sectional	Convenience sampling	NR
[74]	USA	2019	5070	15.8 (1.2)	54.3	School students	Cross-sectional	Convenience sampling	NR
[32]	United Kingdom	2023	252	19.9 (4.7)	NR	University students	Cross-sectional	Convenience sampling	NA
[65]	Saudi Arabia	2023	961	16.7 (2.1)	59.3	School students	Cross-sectional	Convenience sampling	NR
[72]	France	2020	793	33.8 (14.7)	77.3	Adults	Cross-sectional	Convenience sampling	79.0
[73]	Honduras	2022	412	22.2 (4.4)	65.3	University students	Cross-sectional	Convenience sampling	NR
[75]	Australia	2023	273	NR	100	University students	Cross-sectional	Convenience sampling	NA
[33]	Poland	2022	448	24.5 (3.8)	52.2	Adults	Cross-sectional	Convenience sampling	NA
[69]	China	2022	420	19.6 (1.0)	NR	University students	Cross-sectional	Convenience sampling	NR
[71]	Spain	2021	653	14.0 (1.6)	56.0	School students	Cross-sectional	Convenience sampling	NR
[34]	USA	2023	601	20.0 (1.6)	65.7	University students	Cross-sectional	Convenience sampling	NA
[68]	China	2020	3036	16.6 (0.6)	57.0	School students	Cross-sectional	Convenience sampling	NR
[67]	Turkey	2022	1176	15.6 (1.3)	58.4	School students	Cross-sectional	Convenience sampling	NR
[76]	Nigeria	2023	314	NR	NR	University students	Cross-sectional	Convenience sampling	56.4

Note: NA: not applicable; NR: not reported.

**Table 2.** Assessment of TikTok use and mental health variables in the studies that were included in this systematic review.

**Reference**	**Valid scale for the assessment of TikTok use**	**Assessment of TikTok use**	**Mental health variables**	**Valid scale for the assessment of mental health variables**	**Assessment of mental health variables**
[70]	Yes	SAS-SV (adapted version)^a^	Depression Social anxiety Distress intolerance	Yes Yes Yes	PHQ-9 SIAS DIS
[31]	Yes	BSMAS (adapted version)^b^	Depression Anxiety	Yes No	CES-D scale Twenty items
[66]	No	Six items (e.g., I feel irritated, because I feel too responsible for my TikTok friends' fun)	Narcissism	Yes	NPI-16
[74]	No	One item (Do you use TikTok?)	Body image issues	No	One item (Do you have body image issues?)
[32]	Yes	BSMAS (adapted version)^b^	Depression Loneliness	Yes Yes	CES-D scale UCLA Loneliness Scale
[65]	No	One item (Do you use TikTok?)	Poor sleep	Yes	PSQI
[72]	No	One item (Do you use TikTok?)	Life satisfaction	Yes	SWLS
[73]	No	One item (Do you use TikTok?)	Depression Anxiety	Yes Yes	PHQ-9 GAD-7
[75]	No	One item (How often do you use TikTok?)	Disordered eating behavior	Yes	EAT-26
[33]	Yes	BSMAS (adapted version)^b^	Depression	Yes	PHQ-9
[69]	Yes	BSMAS (adapted version)^b^	Anxiety	Yes	STAI
[71]	No	One item (How often do you use TikTok?)	Disordered eating	Yes	SCOFF
[34]	Yes	BSMAS (adapted version)^b^	Depression Loneliness	Yes	PHQ-9 UCLA Loneliness Scale
[68]	Yes	SAS-SV (adapted version)^a^	Depression Anxiety Stress	Yes Yes Yes	DASS-21 DASS-21 DASS-21
[67]	No	One item (Do you use TikTok?)	Loneliness Anger	Yes Yes	UCLA Loneliness Scale AARS
[76]	No	One item (How often do you use TikTok?)	Self-esteem	No	One item (How much self-esteem do you feel?)

Note: AARS: Adolescent Anger Rating Scale; BSMAS: Bergen Social Media Addiction Scale; CES-D Scale: Center for Epidemiologic Studies Depression Scale; DASS-21: Depression Anxiety Stress Scales-21; DIS: Distress Intolerance Scale; EAT-26: Eating Attitudes Test-26; GAD-7: Generalized Anxiety Disorder-7; NPI-16: Narcissistic Personality Inventory-16; PHQ-9: Patient Health Questionnaire-9; PSQI: Pittsburgh Sleep Quality Index; SAS-SV: Smartphone Addiction Scale-Short Version; SCOFF: Sick, Control, One, Fat, Food questionnaire; SIAS: Social Interaction Anxiety Scale; STAI: State-Trait Anxiety Inventory; SWLS: Satisfaction With Life Scale. ^a^ Authors replaced “smartphone” with “TikTok” throughout the scale. ^b^ Authors replaced “social media” with “TikTok” throughout the scale.

#### 3.5.2. Anxiety

We calculated the standardized regression coefficients from unstandardized regression coefficients and other measures of effect referring to the relationship between TikTok use and anxiety. In particular, we calculated standardized regression coefficients from data in four studies [31],[68]–[70].

**Figure 3.**
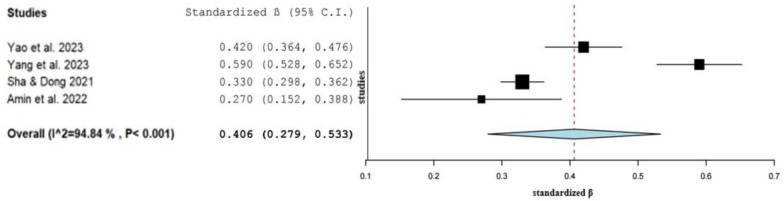
Forest plot of the association between TikTok use and anxiety.

We found a statistically significant positive association between TikTok use and anxiety since the pooled standardized regression coefficient was 0.406 (95% *CI*: 0.279 to 0.533, *p* < 0.001) (Figure 3). Heterogeneity between results was high (*I^2^* = 94.8%, *p*-value for the Hedges *Q* statistic < 0.001). A leave-one-out sensitivity analysis showed that no single study had a disproportional effect on the pooled standardized regression coefficient, which varied between 0.350 (95% *CI*: 0.275 to 0.424, *p* < 0.001), with Yang et al. (2023) [69] excluded, and 0.445 (95% *CI*: 0.297 to 0.593, *p* < 0.001), with Amin et al. (2022) [31] excluded (Supplementary Figure S3). The *p*-value for Egger's test (0.96) and symmetry of the funnel plot (Supplementary Figure S4) indicated an absence of publication bias.

Meta-regression analysis showed that the pooled standardized regression coefficient was stronger for studies carried out more recently (*β* = 0.095, *p* = 0.040). Also, meta-regression showed that an increased percentage of females was associated with an increased pooled standardized regression coefficient (*β* = 0.008, *p* = 0.005). Moreover, the pooled standardized regression coefficient was independent of age (*β* = 0.003, *p* = 0.84) and sample size (*β* = −0.001, *p* = 0.42).

We cannot perform subgroup analysis with the quality of studies as a potential source of heterogeneity since three studies had a moderate risk of bias, and one study had a high risk of bias.

#### 3.5.3. Other mental health variables

Data to perform meta-analysis with the other mental health variables were limited, and, thus, we present findings in a descriptive way. Thus, Williams et al. (2024) [34] found a positive association between TikTok use and loneliness (unstandardized regression coefficient = 0.46, 95% *CI* = 0.31 to 0.60, *p*-value <0.001), but Hendrikse and Limniou (2024) [32] and Sarman and Tuncay (2023) [67] did not find a statistically significant relationship (*p*-value = 0.55, and *p*-value = 0.36, respectively). Moreover, our review suggested a positive association between TikTok use and body image issues, poor sleep, anger, self-esteem, distress intolerance, narcissism, and stress [65],[66],[68],[70],[74],[76]. On the other hand, our findings suggested that there is no association between TikTok use and disordered eating behavior, life satisfaction, and disordered eating [71],[72],[75].

Table 3 shows measures of effect, 95% confidence intervals, and *p*-values between problematic TikTok use and mental health variables.

**Table 3.** Association between problematic TikTok use and mental health variables.

**Reference**	**Mental health variables**	**Unstandardized regression coefficient (95% *CI*, *p*-value)**	**Correlation coefficient (*p*-value)**	**Other measures of effect**	**Level of analysis**
[70]	Depression Social anxiety Distress intolerance		0.40 (<0.01) 0.42 (<0.01) 0.46 (<0.01)		Univariable
[31]	Depression Anxiety	0.37 (NR, 0.03) 0.27 (NR, 0.01)			Univariable
[66]	Narcissism		0.49 (<0.01)		Univariable
[74]	Body image issues			2.01 (1.74 to 2.31, <0.001)^a^	Multivariable
[32]	Depression Loneliness	0.20 (NR, 0.001) NR (NR, 0.55)			Multivariable
[65]	Poor sleep			1.33 (1.01 to 1.77, 0.049)^a^	Multivariable
[72]	Life satisfaction	−0.04 (NR, >0.05)			Univariable
[73]	Depression Anxiety			1.85 (<0.01)^b^ 1.99 (<0.001)^b^	Univariable
[75]	Disordered eating behavior			0.01 (0.31)^c^	Univariable
[33]	Depression	0.26 (0.16 to 0.37, <0.001)	0.38 (<0.001)		Multivariable
[69]	Anxiety		0.59 (NR)		Univariable
[71]	Disordered eating		0.04 (0.19)		Univariable
[34]	Depression Loneliness	0.28 (0.19 to 0.37, <0.001) 0.46 (0.31 to 0.60, <0.001)	0.29 (<0.01) 0.26 (<0.01)		Multivariable
[68]	Depression Anxiety Stress		0.27 (<0.01) 0.33 (<0.01) 0.33 (<0.01)		Univariable
[67]	Loneliness Anger			−0.4 (0.36)^b^ 2.2 (0.03)^b^	Univariable
[76]	Self-esteem	0.33 (NR, <0.001)			Univariable

Note: *CI*: confidence interval; NR: not reported. ^a^ odds ratio (95% *CI*, *p*-value). ^b^ mean score difference on a scale between TikTok users and non-users (*p*-value). ^c^ eta squared (*p*-value).

## 4. Discussion

To the best of our knowledge, this is the first systematic review and meta-analysis that summarizes the research on the association between problematic TikTok use and mental health. We found that problematic TikTok use has a negative association with the mental health of individuals. In particular, the main findings of our meta-analysis indicated that problematic TikTok use has a positive association with depression and anxiety. Moreover, problematic TikTok use was positively associated with other mental health variables (i.e., body image issues, poor sleep, anger, distress intolerance, narcissism, and stress); however, the number of studies was very limited, and, thus, we cannot perform a meta-analysis. On the other hand, our findings suggested that there is no association between TikTok use and disordered eating behavior, life satisfaction, and disordered eating.

We should note the high heterogeneity in effect sizes for our meta-analysis. Our subgroup analysis identified the quality of the studies as a source of heterogeneity since the positive association between TikTok use and depression was stronger among studies with a moderate and high risk of bias than studies with a low risk of bias. Thus, since the quality of the studies can affect the association between TikTok use and mental health, scholars should conduct more valid studies such as longitudinal studies with representative samples to reduce heterogeneity between results. Moreover, of the 16 studies in our review, only six (37.5%) utilized a valid tool (the Bergen Social Media Addiction Scale and Smartphone Addiction Scale-Short Version) to measure and assess problematic TikTok use. Additionally, although these tools are not designed to measure specifically problematic TikTok use but of social media use in general, authors just adapted them for the case of TikTok. Therefore, measurement of problematic TikTok use with valid and specific tools may reduce the heterogeneity in effect sizes among studies. Another possible reason for high heterogeneity in our meta-analyses was different study populations. Specifically, eight studies used samples from university students, five studies used samples from school students, and three studies used samples from the general population. Another issue that could cause high heterogeneity in effect sizes is confounding. Only five out of 16 studies in our review used multivariable models to eliminate confounders in the association between problematic TikTok use and mental health variables. Since confounders usually overestimate effect sizes it is crucial for future studies to reduce confounding.

It is crucial to recognize that the studies in our review were all cross-sectional, thus the direction of the relationship between TikTok use and mental health issues is difficult to ascertain. Problematic use of TikTok may be a result of, rather than a cause of, conditions like depression and anxiety. The connection might be bidirectional, with poor mental health and TikTok use influencing each other [77],[78]. For instance, individuals experiencing depression might be more inclined to use TikTok extensively. Consequently, it is challenging to ascertain whether prolonged or frequent TikTok use leads to poor mental health outcomes, or if TikTok serves as an ineffective coping mechanism for pre-existing mental health problems [79],[80]. As an example, people with depression and low self-esteem might seek validation from TikTok interactions [81],[82]. This could lead to a cycle of rumination and guilt about TikTok use, perpetuated by negative self-perception and low self-efficacy [81],[83]. The easy accessibility of TikTok and its potential for more moderated social behavior during these interactions may lead the depressed population to favor these platforms more than face-to-face encounters [84].

Our meta-analysis identified a positive association between problematic TikTok use and depression. Literature shows similar findings in other social media platforms. In particular, a recent meta-analysis found a positive correlation between problematic Facebook use and depression (pooled correlation coefficient = 0.35), and psychological distress (pooled correlation coefficient = 0.34). Moreover, Marino et al. found that problematic Facebook use is correlated with reduced well-being (pooled correlation coefficient = −0.22) [28]. Similarly, Casale and Banchi identified the positive association between problematic Facebook use and narcissism through a systematic review of seven studies. Authors found that the correlation coefficient between problematic Facebook use and narcissism range from 0.13 to 0.32 [27]. Another recent meta-analysis found similar results since the pooled correlation coefficient between problematic social media use and depression was 0.27, and psychological distress was 0.31 [29]. Ahmed et al. confirmed the positive correlation between problematic social media use and depression (pooled correlation coefficient = 0.30). Additionally, authors identified a negative correlation between problematic social media use and well-being (pooled correlation coefficient = −0.25), and a positive correlation between problematic social media use (i.e., Facebook, Instagram, WhatsApp, Twitter, TikTok, WeChat, and MySpace) and sleep problems (pooled correlation coefficient = 0.19) [24]. Similarly, a systematic review found a positive correlation between online social networking (i.e., Facebook, Twitter, and MySpace) and depression with correlation coefficients ranged between 0.15 and 0.26 among studies [26]. Another recent systematic review found a significant correlation between problematic social media use and symptoms of depression since the correlation coefficients ranged between 0.13 and 0.45 [23].

Research has shown that using social media can negatively impact mood, well-being, and overall life satisfaction [85]–[87]. For example, passively consuming content on social media platforms, rather than actively engaging in communication, has been associated with reduced social connections and increased feelings of loneliness and social isolation [88]. One possible reason for these differences is that idealized depictions of peers on these platforms might incite envy and the false belief that others are leading more fulfilling or successful lives [89]. Over time, these feelings of jealousy could lead to a sense of inferiority and depression [90]. Another potential factor might be the perception of wasting time on trivial social media activities, which could negatively impact one's mood [87]. Additionally, the substantial rise in time spent on social media has led some experts to call for recognizing “internet addiction” as a distinct condition closely associated with depression [91],[92]. Another theory proposes that depression may arise from reduced self-esteem when users unfavorably compare themselves to carefully curated images of seemingly more attractive, slimmer, popular, or wealthy individuals [93],[94]. Finally, increased exposure to social media might heighten the risk of cyber-bullying, potentially contributing to depressive symptoms [95].

TikTok's captivating design, which prompts users to find themselves spending a lot of time scrolling through content, may result in excessive screen time. Research indicates that the flow experience significantly impacts addiction behaviors on TikTok and other social platforms, both directly and indirectly [96],[97]. Evidence suggests that enjoyment positively correlates with concentration, subsequently leading to time distortion and problematic TikTok use [96]. One possible connection between TikTok and depression is the fear of missing out, which has been recognized as a significant factor in increasing anxiety and depressive symptoms [98]. Some TikTok content may cover sensitive topics, such as mental health challenges or struggles. Continuous exposure to this type of content might influence mood and lead to feelings of sadness or hopelessness. Additionally, TikTok can spread misinformation, which may encourage unhealthy beliefs among its users [97]. Like other social media platforms, TikTok promotes social comparison. Users might measure their lives, appearances, and achievements against those displayed on the platform, leading to feelings of inadequacy or low self-esteem, both of which are linked to depression [35],[99]. Furthermore, using TikTok, especially late at night, can interfere with sleep patterns due to bedtime procrastination [100]. This procrastination serves as a mediator in the connection between smartphone addiction and depression, while sleep disturbances are associated with an increased risk of depression [101].

Similarly, we found a positive association between problematic TikTok use and anxiety. Literature is in accordance with our findings since a recent meta-analysis found that problematic social media use and anxiety were positively correlated (pooled correlation coefficient = 0.34) [30]. Shannon et al. (2022) [29] also performed a meta-analysis and they found a statistically significant correlation (pooled correlation coefficient = 0.35). Marino et al. (2018) [28] found similar results in their meta-analysis since the pooled correlation coefficient between problematic Facebook use and anxiety was 0.33. Moreover, Abbouyi et al. (2024) [23] performed a systematic review and found that the correlation coefficients between problematic social media use and anxiety ranged between 0.17 and 0.39.

Fear of missing out is a possible explanation for the association between problematic social media use and psychological distress [102]. Frequent social media engagement can trigger fear of missing out anxiety, which in turn may intensify the urge to check these platforms more frequently [103]. Simultaneously managing multiple social media accounts and striving to stay current with them can induce anxiety, exacerbating fear of missing out and perpetuating a cycle of increased anxiety [104]. Further research have also linked excessive social media use to reduced creativity and a diminished perception of one's intellectual capabilities [105]. Heavy use of social media use can deprive individuals of real-world interactions with peers, potentially leading to feelings of isolation, loneliness, and anxiety [102]. Moreover, individuals struggling with anxiety often prefer online communication due to challenges with face-to-face interactions [106]. Problematic social media use may also increase concerns about negative evaluations and vulnerability to cyberbullying, and foster negative online interactions, all of which can contribute to elevated anxiety levels [30].

The engaging design of TikTok, which encourages users to spend considerable time scrolling through content, may lead to excessive screen time. As TikTok users become accustomed to consuming information through short videos, they often develop a reduced tolerance for longer, more rigorous methods of acquiring knowledge, such as reading books or watching extended, professional-quality videos. Consequently, TikTok users may struggle to focus on assigned reading materials or tasks, resulting in decreased efficiency and productivity. This can reignite anxiety as individuals struggle to adapt to work demands that no longer align with their expectations. Such circumstances lead to disappointment in oneself, potentially leading to feelings of depression, anxiety, and increased stress levels [107].

Although the number of studies to perform meta-analysis with other mental health variables was limited, our review suggests the negative association between problematic TikTok use and body image issues, poor sleep, anger, distress intolerance, narcissism, and stress. Several systematic reviews and meta-analyses support our findings. In particular, a recent meta-analysis found that the pooled correlation coefficient between problematic social media use and stress was 0.31 [29]. Another meta-analysis identified the positive association between problematic Facebook use and psychological distress (pooled correlation coefficient = 0.29) [28]. A recent systematic review found a positive correlation between problematic social media use and poor sleep quality, and a negative correlation between problematic social media use and sleep duration [25]. Another systematic review revealed a positive correlation between problematic Facebook use and narcissism with correlation coefficients ranged between 0.13 and 0.32 in six studies [27]. Two systematic reviews showed the negative association between social media and body image issues [108],[109]. In particular, Mironica et al. (2024) [108] found a link between social media's emphasis on visual aesthetics, and social appearance anxiety, body dissatisfaction, and beauty perception. Additionally, Vincente-Benito and Ramírez-Durán (2023) [109] revealed the association between misuse or intensive social media use and body dissatisfaction and eating disorders. On the contrary, in our review, we identified two studies that investigated the association between problematic TikTok use and eating disorders but they did not find a statistically significant association [71],[75].

In our review, we recognize the absence of a scale to specifically measure problematic TikTok use. Most studies used non-valid self-reported items of problematic TikTok use (e.g., “How often do you use TikTok?”), whereas a few studies used adapted versions of valid scales which were developed to measure problematic social media use in general (e.g., Bergen Social Media Addiction Scale). More recently, a new scale was developed to specifically assess problematic use of TikTok [110]. Particularly, scholars focused their study only on TikTok use and developed the TikTok Addiction Scale (TTAS). Development of the TTAS is based on the six core components of addiction, i.e., salience, mood modification, tolerance, withdrawal, conflict, and relapse. The TTAS is proven to be a reliable and valid tool in a sample of adults. Further studies should examine the validity of the TTAS in different populations, cultures, settings, and languages. In addition, utilizing a dedicated scale to assess problematic TikTok use will provide for more valid conclusions about the association between TikTok and the mental health of individuals.

Additionally, we found no study assessing the prevalence of problematic use of TikTok. In our review, five studies [31]–[34],[69] used adapted versions of the Bergen Social Media Addiction Scale [42] to measure problematic TikTok use, and two studies [68],[70] used adapted versions of the Smartphone Addiction Scale-Short Version [111]. Several studies have suggested cut-off points for the BSMAS [112]–[114] and the SAS-SV [111],[115] to discriminate addicted social media/smartphone users versus non-addicted users, and, thus, estimate the prevalence of social media/smartphone addiction. However, no study so far has classified TikTok users into addicted and non-addicted categories and therefore has not measured the prevalence of TikTok addiction or problematic TikTok use. Perhaps, the lack of a scale that specifically measures TikTok use may prevent scholars from measuring the prevalence of problematic TikTok use or TikTok addiction. As previously stated, the newly developed TTAS specifically measures TikTok use, and the suggested cut-off point [116] by the developers of the scale may help scholars to identify problematic TikTok users. Timely recognition of signs of TikTok addiction is essential to reduce negative implications and improve users' mental health.

Our study has several limitations. First, data to perform meta-analysis are scarce. Specifically, we can perform meta-analysis only for two mental health variables, i.e., depression and anxiety. Subsequently, subgroup analysis is allowed only for some variables. For instance, there are only four studies set in European countries, two studies in the USA, and one study in Australia. Moreover, only one study examined the association between TikTok use and several mental health variables, such as body image issues, poor sleep, anger, distress intolerance, narcissism, and disordered eating. Therefore, we cannot generalize our results since the representativeness of our studies is limited. Scholars should conduct further studies to obtain more valid results. Second, we cannot establish a causal relationship between problematic TikTok use and mental health variables since all studies in our review were cross-sectional. Performing longitudinal studies and measuring TikTok use and mental health variables through time will add strong evidence on this domain. Third, the non-valid measurement of TikTok use in most studies introduces an important information bias. Thus, future studies should use valid tools to measure the levels of TikTok use. Fourth, all studies used convenience samples, and, thus, generalization of our findings should be made with caution. Further studies should be conducted with random and stratified samples to reduce this selection bias. Fifth, only five out of 16 studies used multivariable models to eliminate confounding factors. Since confounders tend to inflate measures of effect, future studies should eliminate confounders to get more valid results of the independent effect of TikTok use on mental health variables. Personality traits (e.g., extraversion, agreeableness, and conscientiousness), resilience, social support, emotional intelligence, and socio-demographic variables (e.g., gender, age, financial status, and educational level) could be considered as potential confounders in future studies. Sixth, our search terms and strategy may not identify all papers that examine TikTok use since we focused on only problematic TikTok use. Future systematic reviews may broaden our research question by using a tighter search strategy. Finally, we searched six major databases to conduct our review by applying an extensive search strategy. Although we applied the PRISMA guidelines, it is still possible that we have failed to include a relevant study in our review. For instance, we limited our search for studies only in the English language.

## 5. Conclusions

Our meta-analysis provides evidence for the positive association between problematic TikTok use and depression and anxiety. Moreover, our descriptive data indicated a positive association between problematic TikTok use and body image issues, poor sleep, anger, distress intolerance, narcissism, and stress. Given the rise in TikTok use, our systematic review and meta-analysis offers insights into how problematic TikTok use may be associated with mental health issues. The observed positive association between problematic TikTok use and depression carries significant implications for future studies and interventions, considering the growing popularity of TikTok and the global burden of depression-related morbidity and mortality. These findings may be valuable to clinicians and public health practitioners. For instance, clinicians might find it beneficial to evaluate TikTok use patterns among depressed patients to identify potentially harmful behaviors that could be exacerbating mood instability. Furthermore, TikTok could be utilized to alleviate the stigma surrounding depression and identify at-risk individuals by detecting self-reported mental health issues on the application. As social media has become an integral part of human communication, it is crucial for clinicians to strike a balance between encouraging positive use and redirecting from problematic behaviors. From a public health perspective, social media platforms like TikTok may offer opportunities for depression screening or disseminating targeted educational content about the condition. Such messages could raise awareness about maladaptive usage and its link to mood disorders.

Additionally, TikTok has become extremely popular in recent years, especially among children and adolescents. Thus, parents and teachers are worried about the negative effects of TikTok on young people. However, moderate use of TikTok might not be so harmful after all. Cutting off children and adolescents from TikTok, which is the most favored social media platform among their friends, could actually harm their relationships and emotional well-being. Keeping feelings bottled up inside can be harmful to mental health. Moreover, the addictive use of TikTok poses a significant threat to users' mental health. It is important to develop ways to help people who are addicted to TikTok and similar apps. This could include interventions like educational programs, digital wellness initiatives, and counseling services. Working together with healthcare professionals, teachers, and families will be crucial in helping people who are addicted to TikTok and supporting their mental health. It is important to develop guidelines and regulations in order to encourage people to use TikTok in a healthy way. By setting limits and promoting responsible use, we can reduce the negative effects of TikTok on mental health.

## Use of AI tools declaration

The authors declare they have not used Artificial Intelligence (AI) tools in the creation of this article.


