# Supplementary material for: Association between problematic TikTok use and mental health: A systematic review and meta-analysis
Source: AIMS Public Health. 2025 Apr 16;12(2):491–519. doi: 10.3934/publichealth.2025027 (PMC12277781; doi:10.3934/publichealth.2025027)
Supplement: Supplementary file 1 [file publichealth-12-02-027-s001.pdf]

AIMS Public Health

AIMS Public Health, 12(2): 491–519.
DOI: 10.3934/publichealth.2025027
Received: 26 December 2024
Revised: 24 March 2025
Accepted: 02 April 2025
Published: 16 April 2025

https://www.aimspress.com/journal/aimsph

Review

Association between problematic TikTok use and mental health: A systematic review and meta-analysis

Petros Galanis[1,*], Aglaia Katsiroumpa[1], Zoe Katsiroumpa[1], Polyxeni Mangoulia[2], Parisis Gallos[3], Ioannis Moisoglou[4] and Evmorfia Koukia[5]

[1] Clinical Epidemiology Laboratory, Faculty of Nursing, National and Kapodistrian University of Athens, Athens, Greece
[2] Faculty of Nursing, National and Kapodistrian University of Athens, Athens, Greece
[3] Faculty of Nursing, University of West Attica, Athens, Greece
[4] Faculty of Nursing, University of Thessaly, Larissa, Greece
[5] Laboratory Nursing Counselling, Faculty of Nursing, National and Kapodistrian University of Athens, Athens, Greece

* **Correspondence:** Email: pegalan@nurs.uoa.gr; Tel: +30002107461471.

Supplementary

Table S1. Quality of cross-sectional studies included in this systematic review.

	(Yao et al., 2023) [1]	(Amin et al., 2022) [2]	(Asad et al., 2022) [3]	(Sagrera et al., 2022) [4]	(Hendrikse & Limniou, 2024) [5]	(Al-Garni et al., 2024) [6]	(Masciantonio et al., 2021) [7]	(Landa-Blanco et al., 2024) [8]
1. Were the criteria for inclusion in the sample clearly defined?	√	X	X	√	√	√	√	√
2. Were the study subjects and the setting described in detail?	√	√	X	√	√	√	√	√
3. Was the exposure measured in a valid and reliable way?	√	√	X	X	√	X	X	X
4. Were objective, standard criteria used for measurement of the condition?	√	√	X	X	√	X	X	X
5. Were confounding factors identified?	X	X	X	√	√	√	X	X
6. Were strategies to deal with confounding factors stated?	X	X	X	√	√	√	X	X
7. Were the outcomes measured in a valid and reliable way?	√	X	√	X	√	√	√	√
8. Was appropriate statistical analysis used?	√	√	√	√	√	√	√	√
**Total quality**	Moderate	Moderate	Poor	Moderate	Good	Moderate	Moderate	Moderate
	(Blackburn & Hogg, 2024) [9]	(Rogowska & Cincio, 2024) [10]	(Yang et al., 2023) [11]	(López-Gil et al., 2023) [12]	(Williams et al., 2024) [13]	(Sha & Dong, 2021) [14]	(Sarman & Tuncay, 2023) [15]	(Nasidi et al., 2024) [15]
1. Were the criteria for inclusion in the sample clearly defined?	√	√	X	√	√	X	√	X
2. Were the study subjects and the setting described in detail?	√	√	X	√	√	X	√	X
3. Was the exposure measured in a valid and reliable way?	X	√	√	X	√	√	X	X
4. Were objective, standard criteria used for measurement of the condition?	X	√	√	X	√	X	X	X
5. Were confounding factors identified?	X	√	X	X	√	X	X	X
6. Were strategies to deal with confounding factors stated?	X	√	X	X	√	X	X	X
7. Were the outcomes measured in a valid and reliable way?	√	√	√	√	√	√	√	X
8. Was appropriate statistical analysis used?	√	√	√	√	√	√	√	√
**Total quality**	Moderate	Good	Moderate	Moderate	Good	Poor	Moderate	Poor

Note: A score of 7–8 points indicates good quality, a score of 4–6 points indicates moderate quality and a score ≤3 indicates poor quality.

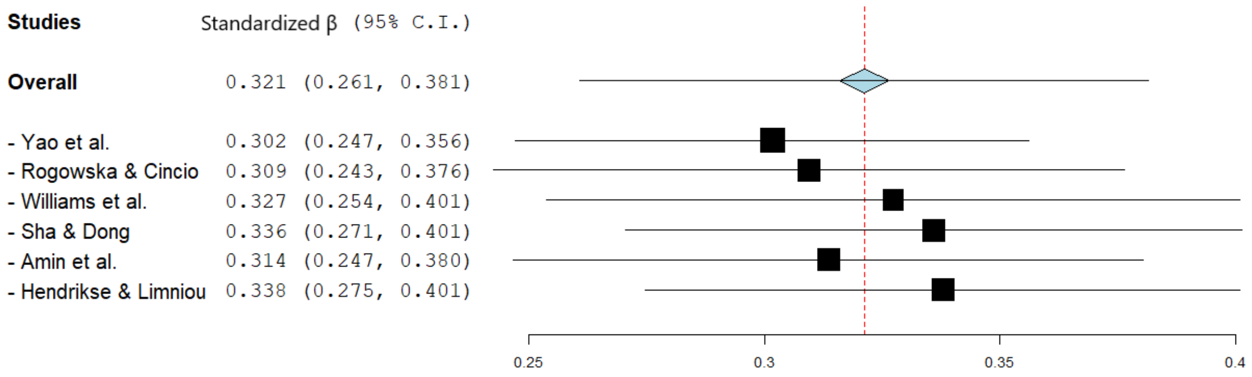

Figure S1. Leave-one-out sensitivity analysis for the association between TikTok use and depression.

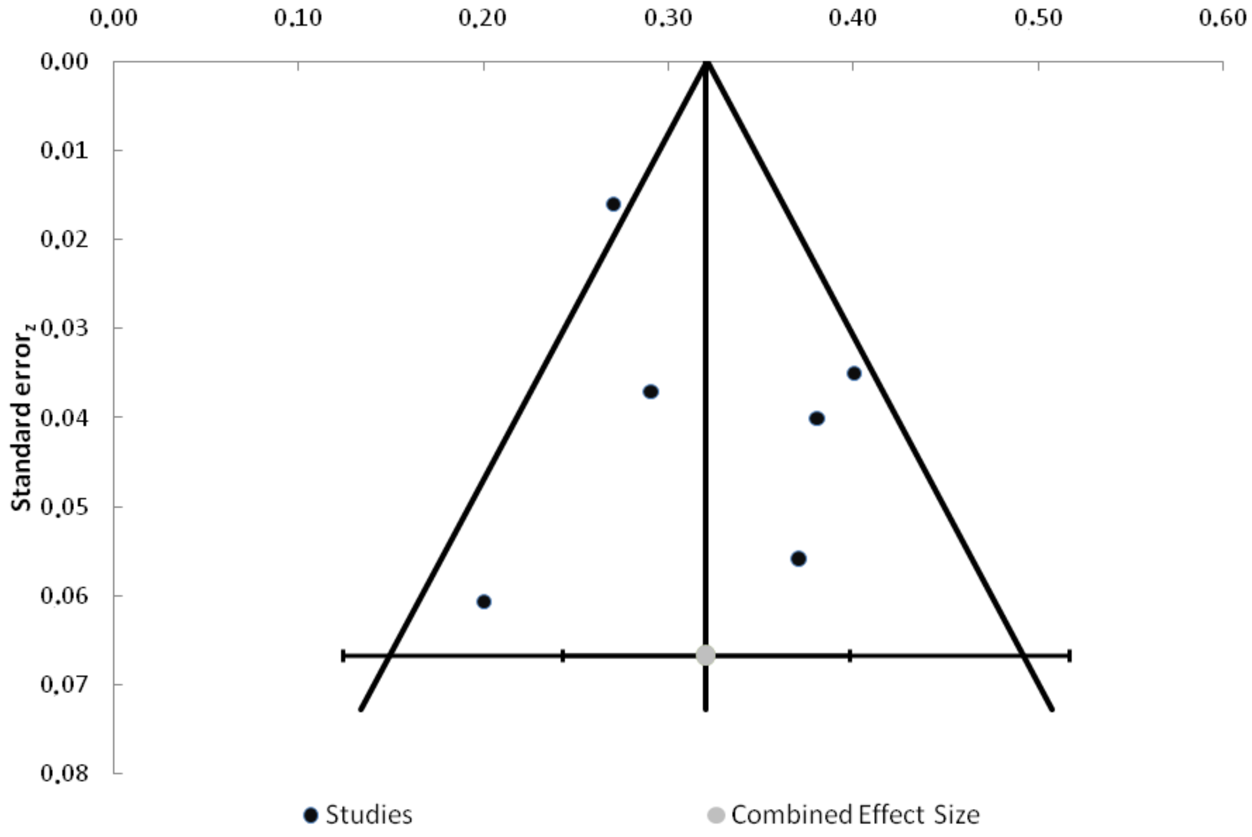

Figure S2. Funnel plot for the association between TikTok use and depression.

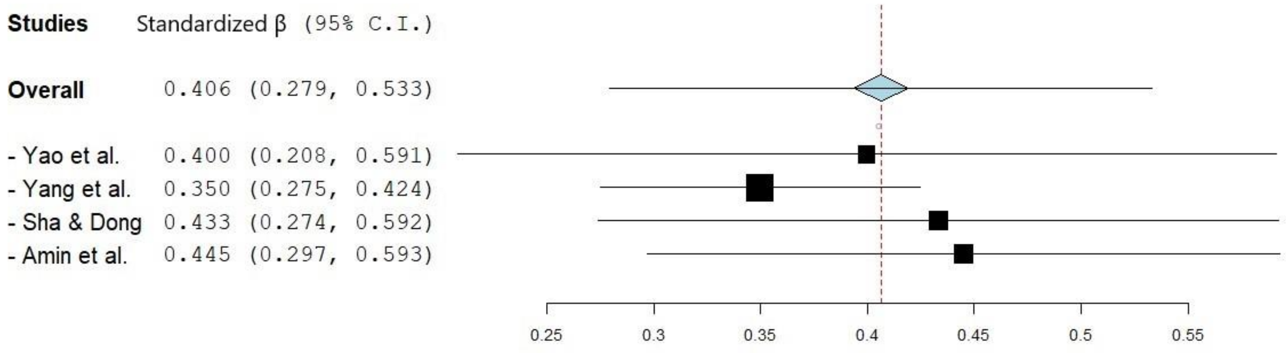

Figure S3. Leave-one-out sensitivity analysis for the association between TikTok use and anxiety.

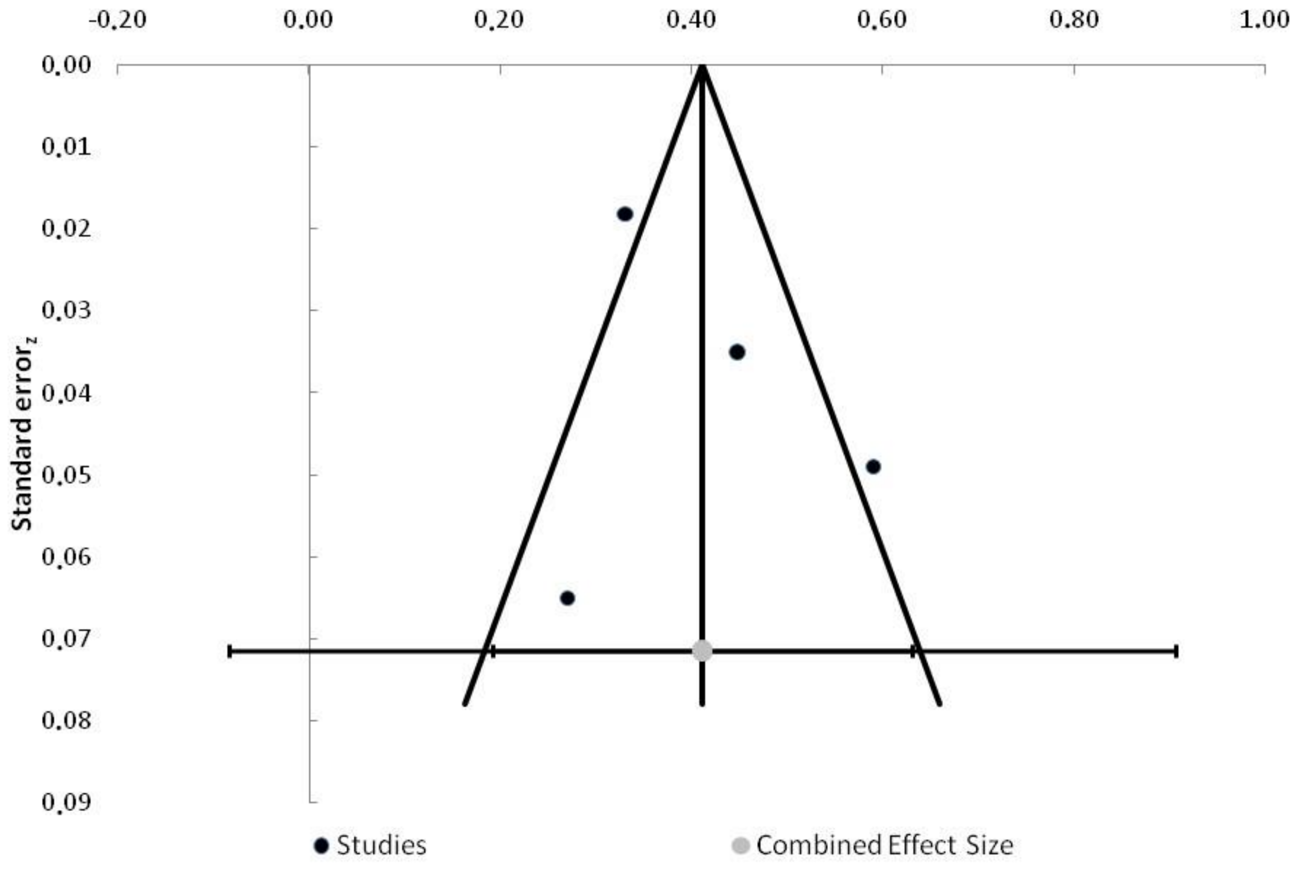

Figure S4. Funnel plot for the association between TikTok use and anxiety.

References

1. Yao N, Chen J, Huang S, et al. (2023) Depression and social anxiety in relation to problematic TikTok use severity: The mediating role of boredom proneness and distress intolerance. *Comput Hum Behav* 145: 107751. https://doi.org/10.1016/j.chb.2023.107751
2. Amin S, Iftikhar A, Meer A (2022) Intervening effects of academic performance between

TikTok obsession and psychological wellbeing challenges in university students. *Online Media Soc* 3: 244–255. https://doi.org/10.71016/oms/qy5har60

3. Asad K, Ali F, Awais M (2022) Personality traits, narcissism and TikTok addiction: A parallel mediation approach. *Int J Media Inf Lit* 7: 293–304. https://doi.org/10.13187/ijmil.2022.2.293
4. Sagrera CE, Magner J, Temple J, et al. (2022) Social media use and body image issues among adolescents in a vulnerable Louisiana community. *Front Psychiatry* 13: 1001336. https://doi.org/10.3389/fpsyt.2022.1001336
5. Hendrikse C, Limniou M (2024) The use of Instagram and TikTok in relation to problematic use and well-Being. *J Technol Behav Sci* 9: 846–857. https://doi.org/10.1007/s41347-024-00399-6
6. Al-Garni AM, Alamri HS, Asiri WMA, et al. (2024) Social media use and sleep quality among secondary school students in Aseer region: A cross-sectional study. *Int J Gen Med* 17: 3093–3106. https://doi.org/10.2147/IJGM.S464457
7. Masciantonio A, Bourguignon D, Bouchat P, et al. (2021) Don't put all social network sites in one basket: Facebook, Instagram, Twitter, TikTok, and their relations with well-being during the COVID-19 pandemic. *PLoS One* 16: e0248384. https://doi.org/10.1371/journal.pone.0248384
8. Landa-Blanco M, García YR, Landa-Blanco AL, et al. (2024) Social media addiction relationship with academic engagement in university students: The mediator role of self-esteem, depression, and anxiety. *Heliyon* 10: e24384. https://doi.org/10.1016/j.heliyon.2024.e24384
9. Blackburn MR, Hogg RC (2024) #ForYou? the impact of pro-ana TikTok content on body image dissatisfaction and internalisation of societal beauty standards. *PLoS One* 19: e0307597. https://doi.org/10.1371/journal.pone.0307597
10. Rogowska AM, Cincio A (2024) Procrastination mediates the relationship between problematic TikTok use and depression among young adults. *J Clin Med* 13: 1247. https://doi.org/10.3390/jcm13051247
11. Yang Y, Adnan H, Sarmiti N (2023) The relationship between anxiety and TikTok addiction among university students in China: Mediated by escapism and use intensity. *Int J Media Inf Lit* 8: 458–464. https://doi.org/10.13187/ijmil.2023.2.458
12. López-Gil JF, Chen S, Jiménez-López E, et al. (2023) Are the use and addiction to social networks associated with disordered eating among adolescents? Findings from the EHDLA study. *Int J Ment Health Addict* 22: 3775–3789. https://doi.org/10.1007/s11469-023-01081-3
13. Williams M, Lewin KM, Meshi D (2024) Problematic use of five different social networking sites is associated with depressive symptoms and loneliness. *Curr Psychol* 43: 20891–20898. https://doi.org/10.1007/s12144-024-05925-6
14. Sha P, Dong X (2021) Research on adolescents regarding the indirect effect of depression, anxiety, and stress between TikTok use disorder and memory loss. *Int J Environ Res Public Health* 18: 8820. https://doi.org/10.3390/ijerph18168820
15. Sarman A, Tuncay S (2023) The relationship of Facebook, Instagram, Twitter, TikTok and WhatsApp/Telegram with loneliness and anger of adolescents living in Turkey: A structural equality model. *J Pediatr Nurs* 72: 16–25. https://doi.org/10.1016/j.pedn.2023.03.017

16. Nasidi QY, Norde AB, Dahiru JM, et al. (2024) Tiktok usage, social comparison, and self-esteem among the youth: Moderating role of gender. *Galactica Media* 6: 121–137. https://doi.org/10.46539/gmd.v6i2.467

AIMS Press

© 2025 the Author(s), licensee AIMS Press. This is an open access article distributed under the terms of the Creative Commons Attribution License (https://creativecommons.org/licenses/by/4.0)
